# Sex-Specific Associations Between Changes in Triglyceride–Glucose (TyG) Index and Risk of Chronic Kidney Disease: A Cohort Study of Young and Middle-Aged Adults

**DOI:** 10.3390/nu17182986

**Published:** 2025-09-17

**Authors:** Yoon Ji Kim, Ye Seul Bae, Yoosoo Chang, Sujeong Shin

**Affiliations:** 1 Big Data Research Center, Division of Healthcare Planning, Kangbuk Samsung Hospital, Sungkyunkwan University School of Medicine, Seoul 03181, Republic of Korea; lizyjk51@gmail.com (Y.J.K.);; 2 Department of Family Medicine, Kangbuk Samsung Hospital, Sungkyunkwan University School of Medicine, Seoul 03181, Republic of Korea; 3 Center of Cohort Studies, Total Healthcare Center, Kangbuk Samsung Hospital, Sungkyunkwan University School of Medicine, Seoul 04514, Republic of Korea; 4 Department of Occupational and Environmental Medicine, Kangbuk Samsung Hospital, Sungkyunkwan University School of Medicine, Seoul 03181, Republic of Korea; 5 Department of Clinical Research Design & Evaluation, SAIHST, Sungkyunkwan University, Seoul 06355, Republic of Korea; 6 Graduate School of Medicine, Sungkyunkwan University, Suwon 16419, Republic of Korea

**Keywords:** triglyceride–glucose index, chronic kidney disease, insulin resistance, young adults, sex differences

## Abstract

**Background/Objectives:** The triglyceride–glucose (TyG) index is a surrogate marker of insulin resistance associated with chronic kidney disease (CKD). The effect of TyG index changes on the development of CKD in young adults remains unclear. **Methods:** We studied 353,140 Korean adults aged 18–49 years who underwent at least two health screenings. The TyG index changes between visits were categorized into quintiles. Incident CKD was defined as an estimated glomerular filtration rate of <60 mL/min/1.73 m^2^ or proteinuria. Cox proportional hazards models were used to estimate the hazard ratios (HRs) for CKD risk. **Results:** Over 2.74 million person-years, 18,857 men and 13,394 women developed CKD. In the fully adjusted models, men in the highest quintile of TyG index increase had a higher CKD risk (HR 1.22, 95% CI 1.16–1.28), while those in the lowest quintile had a lower risk (HR 0.88, 95% CI 0.84–0.92). In women, the associations were in the same direction, but neither the highest (HR 1.01, 95% CI 0.95–1.07) nor the lowest quintile (HR 0.95, 95% CI 0.90–1.00) reached statistical significance. Risk gradients were stronger in those aged 40–49 years, with a baseline eGFR < 90 mL/min/1.73 m^2^, higher alcohol intake, or insulin resistance. **Conclusions:** Among relatively young adults, greater increases in the TyG index were associated with a higher CKD risk in men, whereas decreases were protective. In contrast, no significant associations were observed in women. Monitoring TyG index trajectories may help identify high-risk individuals for early intervention.

## 1. Introduction

Chronic kidney disease (CKD) is increasingly acknowledged as a significant global health concern and is currently the seventh leading cause of mortality worldwide [1]. Although largely preventable, CKD remains under-recognized and under-prioritized in public health [2]. The early stages of CKD are often clinically silent owing to the compensatory capacity of the kidneys [3]. However, timely detection is essential because sustained damage can lead to impaired renal function and progression to end-stage kidney disease (ESKD) [4]. Although the prevalence of CKD is higher in women, men are more likely to progress to ESKD and experience greater mortality [5], reflecting sex-related differences that may involve multiple pathophysiological mechanisms [6]. Therefore, CKD risk assessment and screening should be guided less by chronological age and more by comorbid conditions and individualized risk profiles [7]. Importantly, mildly reduced renal function has also been observed in adults under 50 years of age [8], highlighting this younger population as a high-risk group in which early detection and intervention are warranted. This trend is worsened by the earlier onset of diabetes and cardiovascular diseases, along with poor lifestyle choices, obesity, and unhealthy dietary practices [9]. Early-onset CKD increases the lifetime risk of complications, including cardiovascular morbidity and premature mortality, owing to disease duration [10,11]. This highlights the need for early detection and intervention to reduce long-term health and socioeconomic impact.

Insulin resistance (IR) is recognized for its role in CKD development through pathways such as inflammation, oxidative stress, endothelial dysfunction, and increased glomerular filtration rate. The homeostasis model assessment of insulin resistance (HOMA-IR), which is based on fasting blood glucose (FBG) and insulin levels, is the most widely applied index for assessing IR in clinical practice. However, insulin measurement is costly and not well standardized, which limits its use in primary care and low-resource settings. As an alternative, the triglyceride–glucose (TyG) index was proposed, which could be calculated as ln(fasting triglycerides (mg/dL)×FBG (mg/dL)/2) [12,13]. As the TyG index is calculated solely from FBG and triglyceride values, it is highly cost-effective and easily applicable in large epidemiological studies. Accordingly, the TyG index has emerged as a readily available surrogate marker of IR and has attracted considerable clinical interest [13,14,15]. It also serves as a useful indicator of the underlying pathophysiological process [16,17,18]. Prior studies on the TyG index and CKD have predominantly focused on older populations with substantial comorbidities and relatively small sample sizes. These investigations typically examined either baseline TyG index levels or overall directional changes without addressing transitions across clinically relevant categories [19,20,21,22]. Although some studies have conducted sex-stratified analyses, the reported associations were inconsistent and generally failed to demonstrate clear sex-specific differences [20,21,22], which contrasts with the well-established sex differences in CKD risk. Therefore, it is important to investigate the role of TyG index changes in younger adults and clarify potential sex-specific associations to address a critical knowledge gap in the current literature.

Therefore, this study aimed to investigate the association between changes in the TyG index and CKD incidence in young and middle-aged adults, with particular emphasis on sex-specific differences in this relationship. These findings may provide evidence for early risk stratification of CKD in young populations and inform the development of personalized prevention and management strategies.

## 2. Materials and Methods

### 2.1. Study Population

The Kangbuk Samsung Health Study (KSHS) is a large-scale retrospective cohort study comprising adult men and women who have undergone standardized, comprehensive health examinations on an annual or biennial basis at the Total Healthcare Center of Kangbuk Samsung Hospital in Seoul and Suwon, Republic of Korea, and it has been ongoing since 2002. This cohort was established as part of a health screening examination that included questionnaires, blood tests, and imaging examinations [23].

**Figure 1:**
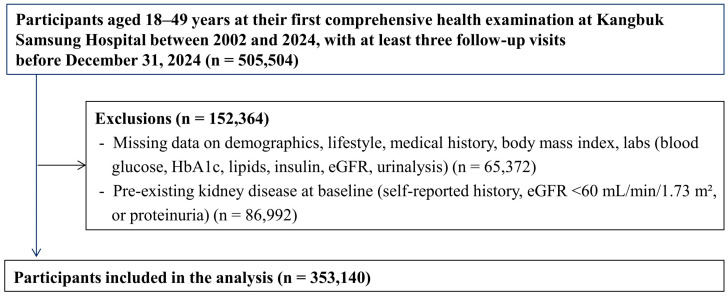
Flowchart of the participants.

This study focused on individuals aged 18–49 years at the time of their first comprehensive health examination at the hospital. A total of 505,504 individuals who underwent at least three health examinations were initially identified and screened for eligibility. Individuals were excluded if they (1) had missing data on key covariates, including age, sex, smoking status, daily alcohol consumption, regular physical activity, education, total cholesterol, high-density lipoprotein cholesterol (HDL-C), low-density lipoprotein cholesterol (LDL-C), triglycerides, FBG, urine albumin, urine acid, estimated glomerular filtration rate (eGFR), body mass index (BMI), use of lipid-lowering medications, history of kidney disease, hypertension, or diabetes, and menopausal status variable for women, or (2) had evidence of kidney disease at baseline, defined as a history of kidney disease, an eGFR < 60 mL/min/1.73 m^2^, or a positive result for urine albumin (proteinuria). After applying the exclusion criteria, the final study population comprised 353,140 individuals (208,312 men and 144,828 women) (Figure 1).

The Kangbuk Samsung Health Study was approved by the Institutional Review Board (IRB) of Kangbuk Samsung Hospital (IRB No. KBSMC 2011-01-030, 7 March 2011). The present sub-study was reviewed and exempted from additional IRB approval (IRB No. KBSMC 2025-09-011) because it used pre-existing, anonymized data. The IRB waived the requirement for informed consent because the analyses were performed on de-identified datasets without any direct participant contact. This study was conducted in accordance with the Declaration of Helsinki and the relevant institutional guidelines and regulations.

### 2.2. Definitions of Exposure and Study Outcome

The exposure variable in this study was the change in the TyG index between the first and second health examinations. The TyG index at each examination was calculated as ln(Triglycerides (mg/dL)×FBG (mg/dL)/2) as previously described. Because no universally accepted clinical cut-off values for the TyG index have been established [18,24,25], participants were categorized into quintiles according to the magnitude of change in the TyG index: Q1 (greatest decrease; −3.420 to −0.295), Q2 (moderate decrease; −0.295 to −0.047), Q3 (no or minimal change; −0.047 to 0.160), Q4 (moderate increase; 0.160 to 0.409), and Q5 (greatest increase; 0.409–3.210).

The outcome of the study was incident chronic kidney disease (CKD), defined as an eGFR of less than 60 mL/min/1.73 m^2^ or the presence of proteinuria during follow-up. Follow-up began at the time of the third health examination and continued until the incidence of CKD, the last available health examination, or 31 December 2024, whichever occurred first. Although the exposure variable was defined as the change between the first and second health examinations, all other covariates used for adjustment were assessed at the time of the first examination and considered baseline characteristics. This approach allowed us to evaluate the effect of TyG index change on incident CKD while controlling for potential confounders that preceded the exposure period.

### 2.3. Measurements

Demographic characteristics (such as age, sex, and education), health behaviors, medical history, and medication use were assessed as a basic part of the health examinations using self-administered questionnaires [26]. Smoking status was categorized as never, former, or current. Alcohol intake was categorized as <20 or ≥20 g/day for men and <10 or ≥10 g/day for women. Regular physical activity was defined as engaging in vigorous physical activity at least three times a week [26,27]. Education was classified into two groups: university education or more and less than university education.

Trained nurses measured the participants’ height and weight with minimal clothing, and blood pressure was measured using an automatic blood pressure monitor after being seated for 10 min. BMI was calculated as weight in kilograms divided by the square of height in meters (kg/m^2^). All blood samples were drawn from the antecubital vein after fasting for a minimum of 10 h. Blood tests included total cholesterol (mg/dL), HDL-C (mg/dL), LDL-C (mg/dL), triglycerides (mg/dL), FBG (mg/dL), insulin (uIU/dL), glycated hemoglobin (HbA1c) (%), and uric acid (mg/dL) levels. The HOMA-IR was calculated as follows: insulin  (µU/mL)×FBG  (mg/dL)/405.

Serum creatinine levels were determined using the kinetic alkaline picrate (Jaffe) method with an automated chemistry analyzer (Modular D2400, Roche, Tokyo, Japan). The estimated glomerular filtration rate (eGFR) was calculated using the Chronic Kidney Disease Epidemiology Collaboration (CKD-EPI) formula. A low eGFR was defined as being less than 60 mL/min/1.73 m^2^, following the Kidney Disease: Improving Global Outcomes (KDIGO) clinical practice guidelines [28]. Since urinary albumin was not assessed, chronic kidney disease (CKD) was defined in the primary analyses as an eGFR below 60 mL/min/1.73 m^2^ and/or the presence of proteinuria. Urine protein was evaluated semi-quantitatively using dipstick tests (URiSCAN Urine Strip, YD Diagnostics, Yong-In, Republic of Korea) on fresh midstream urine samples. The results were graded as negative, trace, 1+, 2+, 3+, or 4+ (equivalent to undetectable, 10, 30, 100, 300, and 1000 mg/dL, respectively). Proteinuria was defined as ≥1+. The eGFR was calculated using the four-variable Modification of Diet in Renal Disease Study equation [27,29].

### 2.4. Statistical Analyses

All analyses were conducted separately for men and women to evaluate sex-specific associations. The baseline characteristics of the study population are presented as mean ± standard deviation (SD) for continuous variables and as numbers (percentages) for categorical variables. Differences across quintiles of TyG index change were assessed using one-way analysis of variance (ANOVA) for continuous variables and chi-square test for categorical variables.

Cox proportional hazards regression models were used to estimate hazard ratios (HRs) and 95% confidence intervals (CIs) for the association between quintiles of TyG index change and the risk of incident CKD. Three models were constructed: (1) age-adjusted model: adjusted for age; (2) multivariable-adjusted model 1: adjusted for age, examination center (Seoul or Suwon), BMI, education, smoking status, alcohol intake, regular physical activity, history of diabetes, history of hypertension, use of lipid-lowering medications, eGFR, TyG index of first examination, and menopausal status (in women); and (3) multivariable-adjusted model 2: adjusted for all variables in Model 1 plus total cholesterol, HDL-C, LDL-C, HOMA-IR, and uric acid. We also performed a sensitivity analysis using the percentage change in the TyG index to evaluate the robustness of the main findings. To further examine potential dose–response relationships, we used restricted cubic spline models with knots at the 5th, 27.5th, 50th, 72.5th, and 95th percentiles of the TyG index distribution for the analysis.

To assess whether the associations differed by sex, fully adjusted Cox models, including the TyG index change quintiles × sex interaction term, were fitted to the entire cohort. The statistical significance of the interaction was evaluated using the likelihood ratio test (LRT), comparing models with and without the interaction term. Post hoc power analyses were conducted using the log-rank test to assess whether the observed HRs could be reliably detected, given the large sample size and number of events. Kaplan–Meier survival curves were constructed to compare the cumulative incidence of CKD across quintiles of TyG index changes. Furthermore, model discrimination was evaluated by calculating receiver operating characteristic (ROC) curves and corresponding area under the curve (AUC) values at 5, 10, and 15 years based on the fully adjusted model. Subgroup analyses were additionally conducted according to baseline eGFR (<90 or ≥90 mL/min/1.73 m^2^), age subgroup (18–29, 30–39, or 40–49 years), regular physical activity (vigorous exercise <3 or ≥3 times/week), alcohol intake (<20 or ≥20 g/day for men and <10 or ≥10 g/day for women), and HOMA-IR (<75th or ≥75th percentile group) [30,31].

Statistical significance was determined by a two-sided *p*-value < 0.05, and all analyses were performed using R software version 4.4.3 (R Core Team, Vienna, Austria) with the packages *survival*, *powerSurvEpi, survminer*, *forestplot,* and *timeROC* [32,33,34,35,36,37].

## 3. Results

### 3.1. Baseline Characteristics

Table 1 and Table 2 present the baseline characteristics of the study population according to the quintiles of TyG index change in men and women. The mean (SD) age of the participants was 34.78 (5.76) years for men and 33.94 (5.84) years for women. The distribution of the TyG index change ranged from −3.420 to 3.210, with quintile cut-off values as follows: Q1 (greatest decrease; −3.420 to −0.295), Q2 (moderate decrease; −0.295 to −0.047), Q3 (no or minimal change; −0.047 to 0.160), Q4 (moderate increase; 0.160 to 0.409), and Q5 (greatest increase; 0.409–3.210). Across both sexes, several cardiometabolic parameters demonstrated consistent patterns. From Q1 to Q5, HDL-C, uric acid, proportion of individuals engaging in regular physical activity, and FBG and triglyceride levels tended to increase. In contrast, age, systolic blood pressure (SBP), diastolic blood pressure (DBP), HOMA-IR, insulin levels, and levels of FBG and triglycerides at the first examination generally decreased across the TyG index change quintiles. Individuals in the highest quintile (Q5) were less likely to have higher educational attainment and more likely to have a history of diabetes and to be taking lipid-lowering medications than those in the lowest quintile (Q1), a pattern observed in both men and women. When examining sex-specific differences, LDL-C showed a gradual increase across TyG index change quintiles, whereas in women, it showed a decreasing pattern (all *p* < 0.001).

A particularly notable observation was the dynamic change in triglyceride and FBG levels between the first and second health examinations. At baseline (first examination), FBG and triglyceride levels were highest in Q1 and lowest in Q5, respectively. However, during the second examination, these patterns were reversed, with Q5 showing the highest level. This reversal indicates that individuals in Q5 experienced a marked increase in glucose and triglyceride levels over time, whereas those in Q1 had substantial reductions, especially in women, where the mean TG and FBG values were nearly halved over time in Q1 and nearly doubled in Q5.

When stratified by quartiles of TyG index levels at the first examination, individuals in the higher quartile exhibited more adverse cardiometabolic profiles in both sexes. These patterns suggest that individuals with an elevated baseline TyG index have significantly worse metabolic risk profiles (Supplementary Tables S1 and S2).

### 3.2. Association Between the Change of TyG Index and Incident CKD

Table 3 shows the association between the quintiles of TyG index change and the risk of incident CKD in men and women. In both sexes, the incidence of CKD increased progressively across the TyG index change quintiles. The median follow-up duration was 6.90 years (IQR: 3.30–11.46 years) for men and 6.27 years (IQR: 3.20–10.18 years) for women, yielding a total of 1,671,122.6 person-years (PY) in men and 1,064,435 PY in women. The corresponding overall incidence rates were 11.28 and 12.58 per 1000 PY for men and women, respectively.

In multivariable-adjusted Model 2, men in the highest TyG index change quintile (Q5) had a significantly higher risk of incident CKD than that of the reference group (Q3) (HR: 1.22, 95% CI: 1.16–1.28, *p* for trend < 0.001), whereas those in the lowest quintile (Q1), reflecting the greatest decrease in TyG index, showed a significantly lower risk (HR: 0.88, 95% CI: 0.84–0.92, *p* for trend < 0.001). In women, the HRs for Q5 (HR: 1.01; 95% CI: 0.95–1.07) and Q1 (HR: 0.95; 95% CI: 0.90–1.00) were not statistically significant, as their 95% CIs included 1. However, the overall trend across quintiles was statistically significant (*p* for trend = 0.032), suggesting a potential dose–response relationship between TyG index change and CKD risk in women. These patterns were generally consistent in the age-adjusted and Model 1 analyses, although the effect sizes were slightly attenuated after full adjustment for confounding factors. The sensitivity analysis based on the percentage change in the TyG index yielded results consistent with those of the quintile-based analyses (Supplementary Table S10). Restricted cubic spline analyses demonstrated a linear dose–response relationship between changes in the TyG index and the risk of incident CKD (Supplementary Figure S1). These findings were consistent with the primary analyses based on the quintile categories.

Importantly, the TyG index change quintiles × sex interaction was statistically significant in the fully adjusted model (*p*-interaction < 0.001), supporting that the association between the TyG index change and CKD incidence differed significantly by sex (Supplementary Table S11).

In post hoc power analyses (Supplementary Table S3), the study had ≥95% power to detect the observed effect sizes in men (Q1 and Q5 vs. Q3), whereas power was limited in women because the observed hazard ratios were very close to unity, resulting in insufficient statistical power despite large sample sizes.

**Figure 2:**
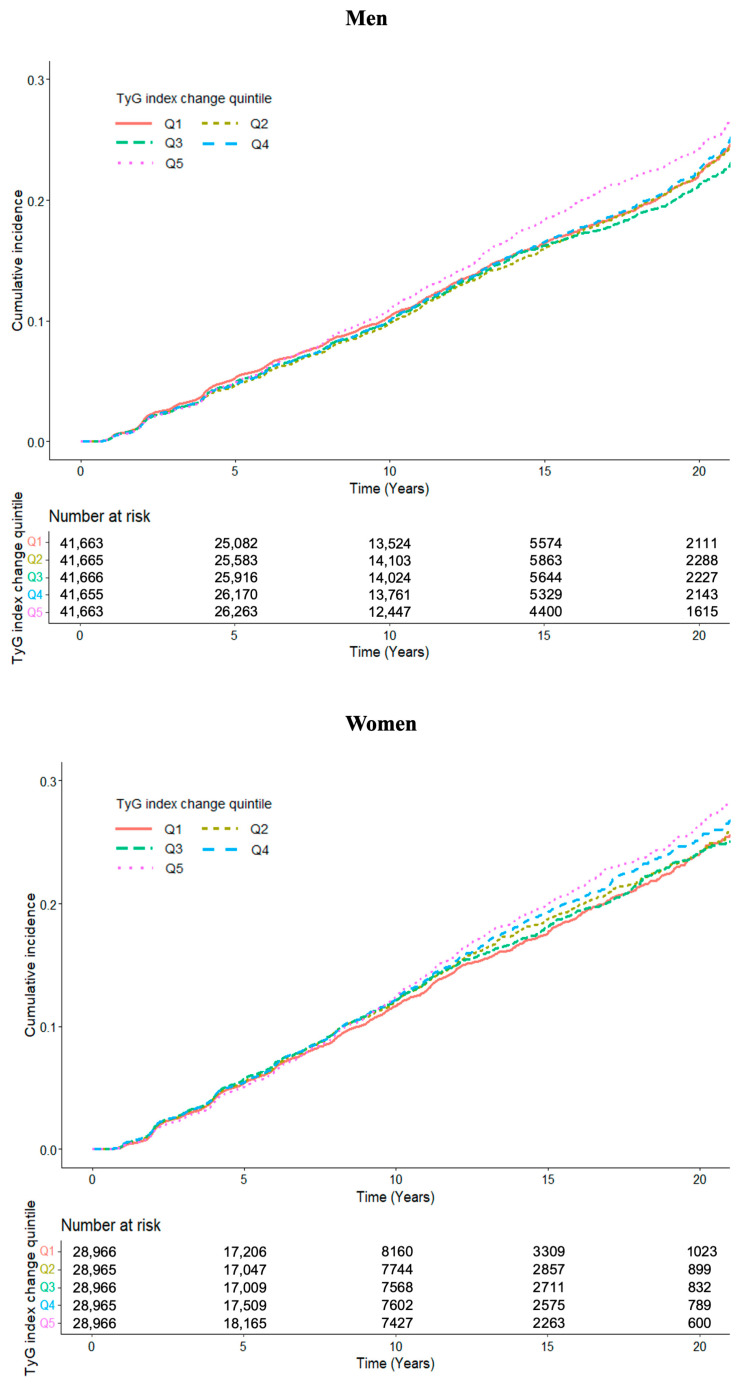
Cumulative incidence of chronic kidney disease according to changes in the triglyceride–glucose (TyG) index, stratified by sex.

These findings suggest that greater increases in the TyG index over time are independently associated with a higher risk of CKD, whereas reductions in the TyG index may confer a protective effect, particularly in men. Kaplan–Meier curves revealed a substantially steeper cumulative incidence curve for Q5 than for the other quintiles, indicating a markedly elevated risk of CKD. This pattern was generally similar for men and women, although the separation between the curves was more pronounced in men (Figure 2).

Supplementary Figure S2 and Supplementary Table S9 show the ROC curves based on a fully adjusted model. The 5-, 10-, and 15-year AUCs were 0.648, 0.682, and 0.683 in men and 0.623, 0.729, and 0.684 in women, respectively. These findings indicate that overall discrimination was modest in both sexes, with slightly higher AUCs observed at longer follow-up periods.

### 3.3. Subgroup Analyses

**Figure 3:**
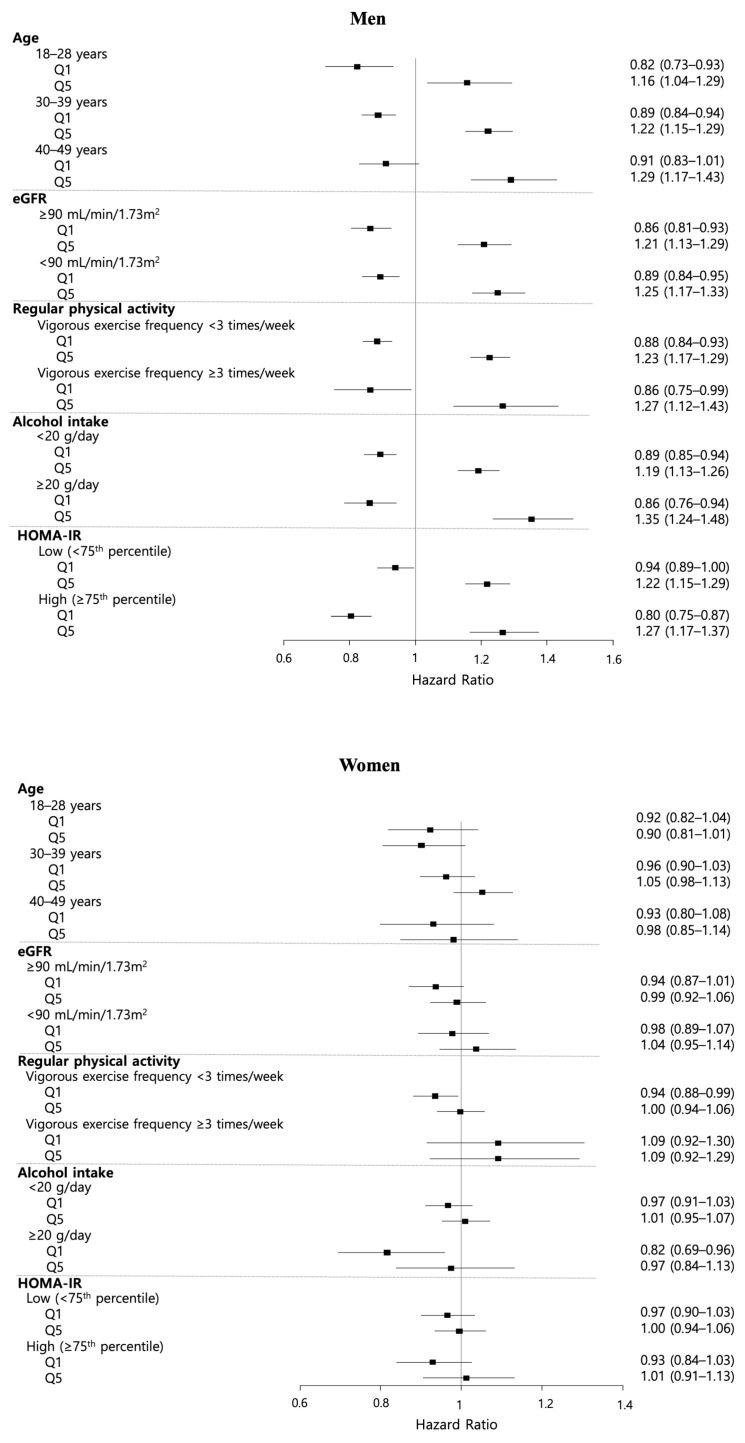
Forest plot of subgroup analyses for chronic kidney disease risk according to triglyceride–glucose (TyG) index changes. Abbreviations. eGRF, estimated glomerular filtration Rate; HOMA-IR, homeostasis model assessment of insulin resistance; Q, quintile.

Subgroup analyses were conducted to further evaluate the association between TyG index change and incident CKD across key baseline characteristics, including baseline age group (18–29, 30–39, or 40–49 years), eGFR level (≥90 or <90 mL/min/1.73 m^2^), regular physical activity status (vigorous exercise <3 or ≥3 times/week), alcohol intake (<20 or ≥20 g/day for men; <10 or ≥10 g/day for women), and HOMA-IR level (<75th or ≥75th percentile) (Supplementary Tables S4–S8). The results are summarized in Figure 3, which shows a forest plot focusing on the extremes of the TyG index change (Q1 and Q5) in each subgroup.

Across most subgroups, particularly among men, there was a consistent trend of increasing CKD risk from Q1 to Q5 for the TyG index change. This indicates that greater increases in the TyG index over time are associated with a higher risk of CKD, independent of baseline metabolic and lifestyle factors.

In men, both the protective effect observed in Q1 and the elevated risk in Q5 were evident in the multivariable analysis. The risk gradient was more pronounced among men aged 40–49 years (HR: 1.29, 95% CI: 1.17–1.43, *p* for trend < 0.001) than among those aged 19–29 years (HR: 1.16, 95% CI: 1.04–1.29), those with baseline eGFR <90 mL/min/1.73 m^2^ (HR: 1.25, 95% CI: 1.17–1.33 vs. ≥90 mL/min/1.73 m^2^: HR: 1.21, 95% CI: 1.13–1.29, *p* for trend < 0.001), and those with higher alcohol intake (HR: 1.35, 95% CI: 1.24–1.48 vs. lower intake: HR: 1.19, 95% CI: 1.13–1.26, *p* for trend < 0.001). In contrast, in women, the HRs for both Q1 and Q5 were 1 across all subgroups, indicating no significant association. However, the point estimates generally trended in the same direction as those observed in men, suggesting a possible but non-significant pattern.

Taken together, these findings indicate that while the upward trend in CKD risk across the TyG index change quintiles is generally consistent, the adverse impact of being in the highest quintile is amplified in subgroups with poorer lifestyle factors and metabolic profiles.

## 4. Discussion

In this large cohort of relatively young adults (<50 years), greater increases in the triglyceride–glucose (TyG) index over time were associated with a higher risk of incident chronic kidney disease (CKD) in men, independent of baseline metabolic and lifestyle factors. Conversely, a reduction in the TyG index was associated with a lower risk of CKD in men. In women, neither an increase nor a decrease in the TyG index was significantly associated with CKD risk, although the direction of the associations was generally similar. These findings highlight that, even in early adulthood and midlife, when the absolute CKD risk is low, within-person changes in the TyG index may identify men at an elevated risk, offering a potential window for early preventive intervention.

Previous studies have shown that higher TyG index levels are associated with increased CKD risk in diverse populations [17,18]. However, most studies relied on single baseline measurements, which may not reflect long-term exposure or capture intra-individual changes affecting disease risk. Recent studies examined TyG index changes in relation to CKD risk, but their designs and populations limited generalizability. Yu et al., in a cohort of hypertensive patients with a mean age in the 60s, classified participants as having either increased or decreased TyG index, failing to capture intermediate patterns [19]. Another study among healthy screening participants with a mean age in the 50s categorized individuals as having persistently high, intermediate, or low TyG index levels, which did not fully reflect within-person improvement or deterioration over time [20]. Evidence regarding apparently healthy young adults remains limited, underscoring the need for research on this population. Our study addressed these gaps by evaluating quintiles of TyG index change in a large cohort of young, healthy adults, enabling a nuanced assessment of CKD risk across the range of change; however, the associations were significant only in men. This temporal approach revealed that risk stratification based on baseline TyG index may fail to identify individuals whose values change substantially over time. This is especially important in younger adults, where early identification of high-risk trajectories may be most impactful. The ability to detect both risk amplification in those with steep increases and risk attenuation in those with decreases offers opportunity for earlier intervention and prevention strategies in patients with diabetes.

Significant associations between changes in the TyG index and incident CKD were observed only in men, while women showed similar trends without statistical significance. This sex-specific pattern reflects differences in metabolic profiles, hormones, and body fat distribution between men and women [38,39]. The TyG index is a validated surrogate marker for hepatic and peripheral insulin resistance, which contributes to CKD pathogenesis through glomerular hyperfiltration, activation of the renin–angiotensin–aldosterone system, endothelial dysfunction, and pro-inflammatory and pro-fibrotic mechanisms [15,17,18]. Progressive insulin resistance exacerbates atherogenic dyslipidemia and oxidative stress, accelerating renal injury [9,40]. Most women in our cohort were premenopausal, with higher estrogen levels that provided vasodilatory, anti-inflammatory, and insulin-sensitizing effects, potentially buffering renal consequences of insulin resistance [41,42]. Men tend to have greater visceral adiposity and adverse cardiometabolic profiles at younger ages, which may magnify the effects of TyG index increases [43,44]. Studies have shown that optimal TyG index cutoffs for insulin resistance and predictive TyG-derived indices for metabolic syndrome differ between sexes [45]. These findings indicate that the TyG index’s interpretation and predictive value vary between sexes. Additionally, another possibility is the insufficient statistical power in women, given the lower absolute incidence of CKD in this group. Moreover, baseline cardiometabolic risk profiles differed substantially by sex, which may have contributed to these divergent results. For instance, in our cohort, the history of hypertension (6.76% vs. 1.36%), diabetes (1.32% vs. 0.45%), and use of lipid-lowering therapy (1.45% vs. 0.36%) were markedly higher in men than in women. These disparities in the underlying risk burden may have amplified the association in men, while attenuating it in women. These physiological and epidemiological differences explain the strong predictive value of TyG index changes for CKD risk in men but not women in our young, healthy cohort. This highlights the need for sex-stratified risk assessment and intervention strategies to identify and prevent CKD, moving beyond a one-size-fits-all approach to incorporating sex-specific metabolic profiles into practice. Future studies should examine if these sex-specific associations persist across populations and investigate biological mechanisms to determine whether modifying TyG index trajectories can reduce CKD risk differently by sex.

A distinctive strength of our study is evaluating TyG index changes in young adults, where CKD is uncommon and under-recognized. We found that TyG index reductions were associated with lower incident CKD risk, while increases predicted higher risk, reflecting metabolic control changes; these associations appeared only in men. Risk gradients were strongest in adults aged 40–49 years, those with baseline eGFR < 90 mL/min/1.73 m^2^, individuals consuming ≥ 20 g alcohol daily, and those in the highest HOMA-IR quartile—indicating that metabolic deterioration has greater renal impact when combined with early midlife, subtle renal impairment, high alcohol intake, or pronounced insulin resistance. These associations’ persistence in high HOMA-IR participants suggests triglycerides may contribute to CKD risk independent of insulin resistance. This is supported by evidence showing triglyceride accumulation in renal cells induces lipotoxicity and inflammation via CD36-mediated lipid uptake, accelerating CKD progression, while interventions modulating triglyceride metabolism can attenuate renal injury [46,47]. These findings highlight the importance of monitoring TyG index trajectories to identify high-risk young adults and guide interventions before irreversible kidney damage occurs. Future studies with lipidomic profiling and trials targeting triglyceride reduction are needed to clarify causal pathways and inform CKD prevention strategies.

Our study had limitations that should be considered in future studies. First, kidney function was assessed using eGFR rather than direct GFR measurement, which may have introduced misclassification, although impractical in large-scale epidemiological research [48]. However, serum creatinine was measured using a standardized enzymatic method at all visits, enhancing internal validity. Second, CKD was defined based on a single measurement of eGFR or proteinuria at each visit without confirmation over ≥3 months, as recommended by guidelines [28], which could have led to over- or underestimation of CKD incidence. Future studies with repeated measurements are needed to confirm persistent kidney dysfunction and minimize misclassification. Third, urine albumin data were unavailable, and proteinuria assessed by dipstick or urine protein–creatinine ratio was used as a surrogate, which may be less sensitive in detecting early kidney damage. Both measures are established prognostic markers of CKD and correlate well with albuminuria in population studies [49]. Fourth, lifestyle factors like smoking, alcohol use, and physical activity were assessed via self-administered questionnaires, which have measurement error and residual confounding, and changes in these factors during follow-up were not considered. Future research should incorporate these as time-varying covariates for more precise assessments. Fifth, we lacked information on specific medications that could affect kidney outcomes or alter triglyceride and glucose levels, such as Sodium-Glucose Cotransporter 2 (SGLT2) inhibitors, Glucagon-Like Peptide-1 (GLP-1) receptor agonists, and fibrates. Future studies should include medication use and duration to better assess the causal relationship between metabolic changes and CKD risk. Sixth, our analysis did not include dietary variables, particularly sugar and fat intakes, which are directly related to the TyG index [50]. The absence of these variables limits our ability to fully account for residual confounding by dietary factors, and this limitation should be considered when interpreting the findings. As with all studies based on routinely collected health screening data, the potential for misclassification of exposure or outcomes cannot be completely excluded. Such non-differential misclassification, if present, would most likely have biased the observed associations toward the null. Therefore, future studies should directly measure and quantify the intake of these nutrients. Finally, our study population consisted of relatively healthy, well-educated, young, and middle-aged Korean adults with good access to healthcare, which may limit the generalizability of our findings. However, the underlying biological mechanisms linking insulin resistance, as reflected by the TyG index, to kidney dysfunction are likely to be broadly relevant. Future studies with more diverse cohorts are warranted to validate and extend our observations.

## 5. Conclusions

In conclusion, in this large cohort of young Korean adults, greater increases in the TyG index over time were independently associated with a higher risk of incident CKD in men, whereas decreases in the TyG index were associated with a lower risk of incident CKD. Although the association in women was not statistically significant, the trends were similar. The risk gradients were particularly pronounced among individuals aged 40–49 years and those with a lower baseline eGFR, higher alcohol intake, and greater insulin resistance. These findings suggest that monitoring TyG index trajectories may provide a practical and early means of identifying young adults at increased risk of CKD, especially those with additional susceptibility factors, and may inform timely interventions to prevent irreversible kidney damage in the future. Further studies are warranted to validate these associations in diverse populations and elucidate the underlying biological mechanisms.

## Figures and Tables

**Table 1:** Baseline characteristics of men by quintiles of triglyceride–glucose (TyG) index changes.

	Total	TyG Index Change Quintiles					
		Q1 (−3.420–−0.295)	Q2 (−0.295–−0.047)	Q3 (−0.047–0.160)	Q4 (0.160–0.409)	Q5 (0.409–3.210)	*p*-Value
N	208,312	41,663	41,665	41,666	41,665	41,663	
Age, years	34.78 ± 5.76	35.29 ± 5.82	35.04 ± 5.76	34.81 ± 5.68	34.58 ± 5.69	34.18 ± 5.76	<0.001
Alcohol intake ≥ 20 g/day (%)	50,184 (24.09)	10,797 (25.92)	9652 (23.17)	9574 (22.98)	9646 (23.16)	10,515 (25.24)	<0.001
Regular physical activity (%) ^a^	30,346 (14.57)	5638 (13.53)	5639 (13.53)	5613 (13.47)	6088 (14.62)	7368 (17.68)	<0.001
Smoking status (%)							<0.001
Never smoker	62,358 (29.93)	12,055 (28.93)	12,198 (29.28)	12,391 (29.74)	12,644 (30.35)	13,070 (31.37)	
Former smoker	70,281 (33.74)	14,627 (35.11)	14,168 (34.00)	13,956 (33.49)	13,876 (33.31)	13.654 (32.77)	
Current smoker	75,673 (36.33)	14,981 (35.96)	15,299 (36.72)	15,319 (36.77)	15,135 (36.33)	14,939 (35.86)	
Education: University or more (%)	161,268 (77.42)	32,160 (77.19)	32,940 (79.06)	32,746 (78.59)	32,557 (78.16)	30,865 (74.08)	<0.001
BMI, kg/m^2^	24.62 ± 3.09	24.94 ± 3.09	24.64 ± 3.09	24.60 ± 3.12	24.51 ± 3.09	24.42 ± 3.03	<0.001
SBP, mmHg	115.06 ± 11.53	116.33 ± 11.88	115.20 ± 11.57	114.89 ± 11.42	114.59 ± 11.36	114.31 ± 11.33	<0.001
DBP, mmHg	73.65 ± 9.29	74.93 ± 9.49	73.92 ± 9.32	73.60 ± 9.21	73.22 ± 9.15	72.59 ± 9.10	<0.001
FBG, mg/dL							
1st examination	95.28 ± 13.65	98.97 ± 19.53	95.80 ± 11.86	94.88 ± 11.34	94.10 ± 11.00	92.68 ± 11.74	<0.001
2nd examination	96.71 ± 14.42	94.64 ± 13.46	95.53 ± 11.77	96.31 ± 12.37	97.23 ± 13.23	99.87 ± 19.39	<0.001
Triglycerides, mg/dL							
1st examination	134.16 ± 88.69	178.55 ± 120.99	142.43 ± 80.82	130.20 ± 74.30	117.68 ± 71.80	101.96 ± 64.21	<0.001
2nd examination	139.98 ± 93.71	103.26 ± 61.04	120.94 ± 68.52	135.59 ± 76.62	150.21 ± 90.82	189.90 ± 130.54	<0.001
TyG index							
1st examination	8.60 ± 0.57	8.93 ± 0.57	8.70 ± 0.52	8.60 ± 0.52	8.49 ± 0.52	8.32 ± 0.55	<0.001
2nd examination	8.66 ± 0.58	8.36 ± 0.54	8.53 ± 0.52	8.65 ± 0.52	8.76 ± 0.52	8.99 ± 0.57	<0.001
Total cholesterol, mg/dL	197.00 ± 34.32	201.15 ± 35.69	197.72 ± 33.82	196.60 ± 33.86	195.38 ± 33.79	194.14 ± 33.99	<0.001
HDL cholesterol, mg/dL	53.85 ± 12.56	52.35 ± 12.33	53.65 ± 12.52	53.85 ± 12.47	54.38 ± 12.56	55.03 ± 12.75	<0.001
LDL cholesterol, mg/dL	125.67 ± 31.54	125.26 ± 31.81	125.11 ± 30.99	125.59 ± 31.30	125.73 ± 31.40	126.67 ± 32.14	<0.001
eGFR, mL/min/1.73 m^2^	92.13 ± 15.71	92.03 ± 16.27	91.51 ± 15.74	91.99 ± 15.76	92.18 ± 15.39	92.94 ± 15.35	<0.001
HbA1c, %	5.49 ± 0.47	5.56 ± 0.65	5.48 ± 0.42	5.47 ± 0.39	5.47 ± 0.38	5.47 ± 0.42	<0.001
Uric acid, mg/dL	6.26 ± 1.22	6.24 ± 1.24	6.22 ± 1.20	6.26 ± 1.21	6.26 ± 1.21	6.35 ± 1.24	<0.001
HOMA-IR	1.79 ± 1.18	2.09 ± 1.38	1.88 ± 1.15	1.79 ± 1.09	1.69 ± 1.08	1.51 ± 1.07	<0.001
Insulin, uIU/mL	7.47 ± 4.29	8.42 ± 4.68	7.82 ± 4.26	7.53 ± 4.15	7.13 ± 4.09	6.44 ± 4.01	<0.001
History of hypertension (%)	14,083 (6.76)	3208 (7.70)	2716 (6.52)	2696 (6.47)	2684 (6.44)	2779 (6.67)	<0.001
History of diabetes (%)	2760 (1.32)	846 (2.03)	462 (1.11)	417 (1.00)	388 (0.93)	647 (1.55)	<0.001
Usage of lipid-lowering medication (%)	3030 (1.45)	672 (1.61)	559 (1.34)	558 (1.34)	591 (1.42)	650 (1.56)	<0.001

Data are expressed as mean ± standard deviation or a number (percentage). One-way ANOVA was used for continuous variables and the chi-square test for categorical variables. BMI, body mass index; DBP, diastolic blood pressure; eGFR, estimated Glomerular Filtration Rate; FBG, fasting blood glucose; HbA1c, glycated hemoglobin; HDL, high-density lipoprotein; HOMA-IR, homeostasis model assessment of insulin resistance; LDL, low-density lipoprotein; Q, quintile; SBP, systolic blood pressure. ^a^ Regular physical activity defined as vigorous exercise frequency ≥ 3 times/week.

**Table 2:** Baseline characteristics of women by quintiles of triglyceride–glucose (TyG) index change.

	Total	TyG Index Change Quintile					
		Q1 (−2.780–−0.297)	Q2 (−0.297–−0.061)	Q3 (−0.061–0.141)	Q4 (0.141–0.381)	Q5 (0.381–3.71)	*p*-Value
N	144,828	28,966	28,965	28,966	28,965	28,966	
Age, years	33.94 ± 5.84	34.13 ± 5.79	34.02 ± 5.77	34.03 ± 5.78	33.98 ± 5.87	33.56 ± 5.98	<0.001
Alcohol intake ≥ 10 g/day (%)	16,609 (11.47)	3227 (11.14)	3150 (10.88)	3266 (11.28)	3238 (11.18)	3728 (12.87)	<0.001
Regular physical activity (%) ^a^	15,169 (10.47)	2851 (9.84)	2887 (9.97)	2889 (9.97)	3037 (10.49)	3505 (12.10)	<0.001
Smoking status (%)							<0.001
Never smoker	130,881 (90.37)	26,075 (90.02)	26,238 (90.59)	26,245 (90.16)	26,289 (90.76)	26,034 (89.88)	
Former smoker	10,405 (7.18)	2162 (7.46)	2107 (7.27)	2014 (6.95)	1951 (6.74)	2171 (7.49)	
Current smoker	3542 (2.45)	729 (2.52)	620 (2.14)	707 (2.44)	725 (2.50)	761 (2.63)	
Education: University or more (%)	93,703 (64.70)	18,347 (63.34)	19,075 (65.86)	19,295 (66.61)	19,064 (65.82)	17,922 (61.87)	
BMI, kg/m^2^	21.59 ± 3.09	21.98 ± 3.26	21.52 ± 3.06	21.43 ± 3.02	21.40 ± 3.01	21.60 ± 3.05	<0.001
SBP, mmHg	102.20 ± 11.02	104.04 ± 11.40	103.07 ± 11.10	102.68 ± 10.98	102.36 ± 10.81	102.37 ± 10.69	<0.001
DBP, mmHg	65.68 ± 8.25	66.41 ± 8.50	65.84 ± 8.27	65.53 ± 8.21	65.34 ± 8.14	65.30 ± 8.09	<0.001
FBG, mg/dL							
1st examination	90.48 ± 10.22	92.70 ± 12.52	91.19 ± 9.72	90.44 ± 8.91	89.52 ± 9.28	88.56 ± 9.78	<0.001
2nd examination	91.23 ± 10.93	89.17 ± 9.99	90.25 ± 9.69	91.14 ± 9.44	91.97 ± 10.01	93.61 ± 14.24	<0.001
Triglycerides, mg/dL							
1st examination	82.08 ± 46.00	111.23 ± 63.64	86.08 ± 41.62	77.21 ± 36.37	71.22 ± 33.69	64.63 ± 32.06	<0.001
2nd examination	85.54 ± 49.61	65.12 ± 31.06	73.05 ± 34.36	79.66 ± 36.80	89.49 ± 42.61	120.38 ± 71.80	<0.001
TyG index							
1st examination	8.11 ± 0.47	8.43 ± 0.46	8.19 ± 0.41	8.07 ± 0.41	7.98 ± 0.42	7.87 ± 0.44	<0.001
2nd examination	8.15 ± 0.48	7.88 ± 0.43	8.02 ± 0.41	8.11 ± 0.41	8.24 ± 0.42	8.51 ± 0.48	<0.001
Total cholesterol, mg/dL	184.21 ± 31.22	187.90 ± 33.35	184.27 ± 30.84	183.26 ± 30.08	182.82 ± 30.31	182.79 ± 31.12	<0.001
HDL cholesterol, mg/dL	66.87 ± 15.28	64.35 ± 15.38	66.57 ± 15.12	67.64 ± 15.13	67.81 ± 15.11	68.00 ± 15.37	<0.001
LDL cholesterol, mg/dL	108.51 ± 28.39	110.55 ± 29.68	108.12 ± 28.07	107.51 ± 27.49	107.67 ± 27.73	108.69 ± 28.84	<0.001
eGFR, mL/min/1.73 m^2^	102.26 ± 21.76	102.11 ± 23.26	101.89 ± 21.88	101.98 ± 21.39	102.04 ± 21.14	103.31 ± 21.05	<0.001
HbA1c, %	5.41 ± 0.35	5.44 ± 0.43	5.40 ± 0.33	5.40 ± 0.32	5.40 ± 0.33	5.41 ± 0.34	<0.001
Uric acid, mg/dL	4.27 ± 0.89	4.24 ± 0.90	4.23 ± 0.88	4.24 ± 0.87	4.28 ± 0.88	4.38 ± 0.93	<0.001
HOMA-IR	1.54 ± 1.24	1.80 ± 1.18	1.60 ± 1.48	1.51 ± 1.43	1.43 ± 0.98	1.35 ± 0.98	<0.001
Insulin, uIU/mL	6.75 ± 4.76	7.72 ± 4.27	6.99 ± 5.84	6.66 ± 5.36	6.34 ± 3.95	6.01 ± 3.83	<0.001
History of hypertension (%)	1970 (1.36)	447 (1.54)	367 (1.27)	388 (1.34)	359 (1.24)	409 (1.41)	0.012
History of diabetes (%)	656 (0.45)	176 (0.61)	103 (0.36)	96 (0.33)	112 (0.39)	169 (0.58)	<0.001
Usage of lipid-lowering medication (%)	524 (0.36)	117 (0.40)	106 (0.37)	75 (0.26)	84 (0.29)	142 (0.49)	<0.001
Menopause (%)	3547 (2.45)	775 (2.68)	658 (2.27)	717 (2.48)	692 (2.39)	705 (2.43)	0.032

Data are expressed as the mean ± standard deviation or number (percentage). One-way ANOVA was used for continuous variables and the chi-square test for categorical variables. BMI, body mass index; DBP, diastolic blood pressure; eGFR, estimated Glomerular Filtration Rate, FBG, fasting blood glucose; HbA1c, glycated hemoglobin; HDL, high-density lipoprotein; HOMA-IR, homeostasis model assessment of insulin resistance; LDL, low-density lipoprotein; Q, quintile; SBP, systolic blood pressure. ^a^ Regular physical activity defined as vigorous exercise frequency ≥ 3 times/week.

**Table 3:** Risk of incident chronic kidney disease according to quintiles of triglyceride–glucose (TyG) index change.

Quintile	N	Events (N)	Duration (PY)	Incidence Rate (per 10^3^ PY)	Age-Adjusted HR (95% CI)	Multivariable-Adjusted HR (95% CI)	
						Model 1 ^a^	Model 2 ^b^
Men							
Q1 (−3.420–−0.295)	41,663	3790	332,099.1	11.41	1.03 (0.99, 1.08)	0.89 (0.85, 0.93)	0.88 (0.84, 0.92)
Q2 (−0.295–−0.047)	41,665	3724	338,970.1	10.99	1.00 (0.95, 1.04)	0.96 (0.92, 1.01)	0.96 (0.92, 1.01)
Q3 (−0.047–0.160)	41,666	3711	338,825.4	10.95	reference	reference	reference
Q4 (0.160–0.409)	41,655	3767	336,736.2	11.19	1.03 (0.98, 1.08)	1.07 (1.02, 1.12)	1.07 (1.02, 1.12)
Q5 (0.409–3.210)	41,663	3865	324,491.8	11.91	1.12 (1.07, 1.17)	1.22 (1.16, 1.27)	1.22 (1.16, 1.28)
*p* for trend					<0.001	<0.001	<0.001
Women							
Q1 (−2.780–−0.297)	28,966	2661	218,342.8	12.19	0.96 (0.91, 1.02)	0.93 (0.88, 0.98)	0.95 (0.90, 1.00)
Q2 (−0.297–−0.061)	28,965	2665	212,029.9	12.57	1.00 (0.95, 1.05)	0.99 (0.94, 1.05)	1.00 (0.95, 1.06)
Q3 (−0.061–0.141)	28,966	2639	210,246.8	12.55	reference	reference	reference
Q4 (0.141–0.381)	28,965	2717	211,568.0	12.84	1.02 (0.97, 1.08)	1.03 (0.98, 1.09)	1.03 (0.98, 1.09)
Q5 (0.381–3.71)	28,966	2708	212,247.5	12.76	1.02 (0.96, 1.07)	1.01 (0.96 1.07)	1.01 (0.95, 1.07)
*p* for trend					0.031	0.002	0.032

Median (Q1–Q3) follow-up duration: 6.90 (3.30–11.46) years for men and 6.27 (3.20–10.18) years for women. Abbreviations: CI, confidence interval; HR, hazard ratio; PY, person-years; Q, quintile. ^a^ Model 1 was adjusted for age, examination center, body mass index, education, smoking status, alcohol intake, regular physical activity, history of diabetes, history of hypertension, use of lipid-lowering medication, eGFR, TyG index, and menopause (for women). ^b^ Model 2 was adjusted for the variables in Model 1, total cholesterol, high-density lipoprotein cholesterol, low-density lipoprotein cholesterol, HOMA-IR, and uric acid.

## Data Availability

The data that support the findings of this study are not publicly available due to institutional policies and ethical restrictions related to the use of personal health information. However, de-identified data may be made available to qualified researchers upon reasonable request and approval by the Institutional Review Board of Kangbuk Samsung Hospital and the Data Sharing Committee of the Kangbuk Samsung Health Study. Requests for data access should be directed to the corresponding author.

## Supplementary Materials

The following supporting information can be downloaded at: https://www.mdpi.com/article/10.3390/nu17182986/s1. Table S1: Baseline characteristics of men by quartiles of TyG index in the first examination. Table S2: Baseline characteristics of women by TyG index quartiles in the first examination. Table S3: Post hoc power analysis for TyG index change quintiles in relation to incident CKD using the log-rank test by sex. Table S4: Risk of incident CKD according to quintiles of TyG index change, stratified by baseline age. Table S5: Risk of incident CKD according to the quintiles of TyG index change by eGFR level. Table S6: Risk of incident CKD according to quintiles of TyG index change, stratified by regular physical activity status. Table S7: Risk of incident CKD according to quintiles of TyG index change, stratified by alcohol intake status. Table S8: Risk of incident CKD according to quintiles of TyG index change, stratified by HOMA-IR group. Table S9: Discrimination ability of TyG index change for chronic kidney disease prediction by time horizon. Table S10: Risk of incident chronic kidney disease according to quintiles of triglyceride–glucose (TyG) index percent change. Table S11: Interaction between TyG index change quintiles and sex in incident chronic kidney disease. Figure S1: Restricted cubic spline curves for the association between changes in the triglyceride–glucose (TyG) index and the risk of incident chronic kidney disease (CKD). Hazard ratios (solid lines) and 95% confidence intervals (shaded areas) were estimated using Cox proportional hazards models with knots at the 5th, 27.5th, 50th, 72.5th, and 95th percentiles of the TyG index change distribution. The models were adjusted for covariates, as described in the Materials and Methods section. Figure S2: Receiver operating characteristic (ROC) curves of men (left) and women (right) at 5, 10, and 15 years. Abbreviations. AUC, area under the curve.

## Abbreviations

The following abbreviations are used in this manuscript:

TyG	Triglyceride–glucose
CKD	Chronic kidney disease
HR	Hazard ratio
CI	Confidence interval
ESKD	End-stage kidney disease
IR	Insulin resistance
KSHS	Kangbuk Samsung Health Study
Q	quintile
HDL–C	High-density lipoprotein cholesterol
LDL-C	Low-density lipoprotein cholesterol
FBG	Fasting blood glucose
eGFR	Estimated glomerular filtration rate
BMI	Body mass index
HbA1c	Glycated hemoglobin
HOMA-IR	Homeostasis model assessment of insulin resistance
SD	Standard deviation
ANOVA	Analysis of variance
LRT	Likelihood ratio test
ROC	Receiver operating characteristic
AUC	Area under the curve
SBP	Systolic blood pressure
DBP	Diastolic blood pressure
PY	Person-year
