# Supplementary material for: Sex-Specific Associations Between Changes in Triglyceride–Glucose (TyG) Index and Risk of Chronic Kidney Disease: A Cohort Study of Young and Middle-Aged Adults
Source: Nutrients. 2025 Sep 17;17(18):2986. doi: 10.3390/nu17182986 (PMC12472455; doi:10.3390/nu17182986)
Supplement: Supplementary file 1 [file nutrients-17-02986-s001.zip › nutrients-3855828-supplementary.pdf]

Supplementary Tables

Supplementary Table S1. Baseline characteristics of men by quartiles of TyG index in the first examination

	Total	TyG index quartile				*p*-value
		**Q1 (5.05 – 8.20)**	**Q2 (8.20 – 8.57)**	**Q3 (8.57 – 8.97)**	**Q4 (8.97 – 13.00)**	
N	208,312	52,157	51,999	52,097	52,059	
Age, years	34.78±5.76	33.07±5.42	34.30±5.62	35.29±5.71	36.45±5.71	<0.001
Alcohol intake≥20g/day (%)	50,184 (24.09)	9,328 (17.88)	11,097 (21.34)	12,948 (24.85)	16,811 (32.29)	<0.001
Regular physical activity (%)[a]	30,346 (14.57)	10,312 (19.77)	7,858 (15.11)	6,616 (12.70)	5,560 (10.68)	<0.001
Smoking status (%)						<0.001
Never smoker	62,358 (29.93)	20,760 (39.80)	16,795 (32.30)	13,999 (26.87)	10,804 (20.75)	
Former smoker	70,281 (33.74)	17,478 (33.51)	17,810 (34.25)	17,928 (34.41)	17,065 (32.78)	
Current smoker	75,673 (36.33)	13,919 (26.69)	17,394 (33.45)	20,170 (38.72)	24,190 (46.47)	
Education: University or more (%)	161,268 (77.42)	40,662 (77.96)	41,140 (79.120	40,762 (78.24)	38,704 (74.35)	<0.001
BMI, kg/m^2	24.62±3.09	23.13±2.59	24.09±2.82	25.05±2.96	26.22±3.08	<0.001
SBP, mmHg	115.06±11.53	111.77±10.46	113.88±10.94	115.86±11.37	118.75±12.13	<0.001
DBP, mmHg	73.65±9.29	70.14±8.34	72.59±8.71	74.61±9.02	77.27±9.53	<0.001
FBG, mg/dL	95.28±13.65	89.74±7.55	93.39±7.62	95.66±9.09	102.35±21.53	<0.001
Triglycerides, mg/dL	134.16±88.69	62.22±12.82	95.71±12.14	135.42±18.65	243.40±110.55	<0.001
Total cholesterol, mg/dL	197.00±34.32	180.23±29.35	192.07±30.56	202.03±32.20	213.68±35.65	<0.001
HDL cholesterol, mg/dL	53.85±12.56	61.40±13.01	55.80±11.70	51.62±10.58	46.58±9.70	<0.001
LDL cholesterol, mg/dL	125.67±31.54	112.33±28.19	124.11±29.17	132.50±30.49	133.78±33.32	<0.001
eGFR, mL/min/1.73m^2	92.13±15.71	94.61±14.92	92.50±15.12	91.04±15.52	90.36±16.86	<0.001
HbA1c, %	5.49±0.47	5.38±0.26	5.43±0.29	5.49±0.35	5.69±0.76	<0.001
Uric acid, mg/dL	6.26±1.22	5.99±1.14	6.13±1.15	6.33±1.20	6.61±1.30	<0.001
HOMA-IR	1.79±1.18	1.16±0.67	1.56±0.82	1.91±1.02	2.54±1.53	<0.001
Insulin, uIU/mL	7.47±4.29	5.15±2.82	6.72±3.36	8.03±3.97	9.97±5.12	<0.001
History of hypertension (%)	14,083 (6.76)	2,046 (3.92)	2,773 (5.33)	3,730 (7.16)	5,534 (10.63)	<0.001
History of diabetes (%)	2,760 (1.32)	214 (0.41)	300 (0.58)	526 (1.01)	1,720 (3.30)	<0.001
Usage of lipid-lowering medication (%)	3,030 (1.45)	362 (0.69)	522 (1.00)	777 (1.49)	1,369 (2.63)	<0.001

Data are expressed as mean±standard deviation or a number (percentage). One-way ANOVA for continuous variables and Chi-square test for categorical variables.

BMI: body mass index; SBP: systolic blood pressure, DBP: diastolic blood pressure, FBG: fasting blood glucose, TyG index: triglyceride and glucose index, HDL: high-density lipoprotein; LDL: low-density lipoprotein, eGFR: estimated Glomerular Filtration Rate, HbA1c: glycated hemoglobin, HOMA-IR: homeostasis model assessment of insulin resistance.

[a]Regular physical activity defined as vigorous exercise frequency $\geq$3times/week.

Supplementary Table S2. Baseline characteristics of women by TyG index quartiles in the first examination

	Total	TyG index quartile				*p*-value
		Q1 (6.14 – 7.78)	Q2 (7.79 – 8.07)	Q3 (8.07 – 8.38)	Q4 (8.38 – 11.80)	
N	144,828	36,338	36,092	36,230	36,168	
Age, years	33.94±5.84	32.76±5.48	33.48±5.64	34.22±5.79	35.32±6.13	<0.001
Alcohol intake≥10g/day (%)	16,609 (11.47)	3,948 (10.86)	3,947 (10.94)	4,122 (11.38)	4,592 (12.70)	<0.001
Regular physical activity (%)[a]	15,169 (10.47)	4,380 (12.05)	3,676 (10.19)	3,566 (9.84)	3,547 (9.81)	<0.001
Smoking status (%)						<0.001
Never smoker	130,881 (90.37)	33,261 (91.53)	32,850 (91.02)	32,744 (90.38)	32,026 (88.55)	
Former smoker	10,405 (7.18)	2,411 (6.63)	2,466 (6.83)	2,613 (7.21)	2,915 (8.06)	
Current smoker	3,542 (2.45)	666 (1.83)	776 (2.15)	873 (2.41)	1,227 (3.39)	
Education: University or more (%)	93,703 (64.70)	25,002 (68.80)	24,470 (67.80)	23,629 (65.22)	20,602 (56.96)	<0.001
BMI, kg/m^2	21.59±3.09	20.53±2.22	20.95±2.51	21.55±2.88	23.32±3.76	<0.001
SBP, mmHg	102.20±11.02	99.84±9.33	101.45±9.98	103.22±10.74	107.12±12.41	<0.001
DBP, mmHg	65.68±8.25	63.57±7.35	64.79±7.61	65.96±8.06	68.43±9.11	<0.001
FBG, mg/dL	90.48±10.22	85.83±7.16	89.26±6.56	91.38±7.04	95.48±15.01	<0.001
Triglycerides, mg/dL	82.08±46.00	45.71±7.55	62.60±6.47	81.62±9.32	138.51±58.26	<0.001
Total cholesterol, mg/dL	184.21±31.22	174.19±27.58	179.94±28.40	185.36±29.69	197.48±33.95	<0.001
HDL cholesterol, mg/dL	66.87±15.28	73.19±14.59	69.60±14.31	65.96±14.10	58.71±14.24	<0.001
LDL cholesterol, mg/dL	108.51±28.39	97.44±23.85	104.25±25.13	110.52±26.93	121.87±31.25	<0.001
eGFR, mL/min/1.73m^2	102.26±21.76	104.50±20.49	102.56±20.94	101.58±21.70	100.41±23.60	<0.001
HbA1c, %	5.41±0.35	5.35±0.26	5.38±0.27	5.40±0.28	5.52±0.53	<0.001
Uric acid, mg/dL	4.27±0.89	4.20±0.86	4.18±0.84	4.24±0.86	4.47±0.97	<0.001
HOMA-IR	1.79±1.18	1.05±0.61	1.34±0.72	1.58±1.16	2.19±1.79	<0.001
Insulin, uIU/mL	6.75±4.76	4.88±2.69	6.01±3.11	6.96±5.00	9.14±6.23	<0.001
History of hypertension (%)	1,970 (1.36)	240 (0.66)	307 (0.85)	456 (1.26)	967 (2.67)	<0.001
History of diabetes (%)	656 (0.45)	59 (0.16)	52 (0.14)	76 (0.21)	469 (1.30)	<0.001
Usage of lipid-lowering medication (%)	524 (0.36)	67 (0.18)	69 (0.19)	135 (0.37)	253 (0.70)	<0.001
Menopause (%)	3,547 (2.45)	682 (1.88)	695 (1.93)	827 (2.28)	1,343 (3.71)	<0.001

Data are expressed as mean±standard deviation or a number (percentage). One-way ANOVA for continuous variables and Chi-square test for categorical variables.

BMI: body mass index; SBP: systolic blood pressure, DBP: diastolic blood pressure, FBG: fasting blood glucose, TyG index: triglyceride and glucose index,

HDL: high-density lipoprotein; LDL: low-density lipoprotein, eGFR: estimated Glomerular Filtration Rate, HbA1c: glycated hemoglobin, HOMA-IR: homeostasis model assessment of insulin resistance.

[a]Regular physical activity defined as vigorous exercise frequency ≥3times/week.

Supplementary Table S3. Post-hoc power analysis for TyG index change quintiles in relation to incident CKD using the log-rank test by sex

Quintile	n	Events (n)	Multivariable-adjusted HR (Model 2)	Post-hoc power
Men				
Q1 (-3.420 – -0.295)	41,663	3,790	0.88 (0.84, 0.92)	≥95%
Q2 (-0.295 – -0.047)	41,665	3,724	0.96 (0.92, 1.01)	42.1%
Q3 (-0.047 – 0.160)	41,666	3,711	reference	reference
Q4 (0.160 – 0.409)	41,655	3,767	1.07 (1.02, 1.12)	83.3%
Q5 (0.409 – 3.210)	41,663	3,865	1.22 (1.16, 1.28)	≥95%
p for trend			<0.001	
Women				
Q1 (-2.780 – -0.297)	28,966	2,661	0.95 (0.90, 1.00)	46.3%
Q2 (-0.297 – -0.061)	28,965	2,665	1.00 (0.95, 1.06)	≤5%
Q3 (-0.061 – 0.141)	28,966	2,639	reference	reference
Q4 (0.141 – 0.381)	28,965	2,717	1.03 (0.98, 1.09)	19.0%
Q5 (0.381 – 3.71)	28,966	2,708	1.01 (0.95, 1.07)	5.5%
p for trend			0.032	

CKD: chronic kidney disease. TyG index: triglyceride and glucose index. HR: hazard ratio.

Supplementary Table S4. Risk of incident CKD according to quintiles of TyG index change, stratified by baseline age

	Quintile	Events (n)	Duration (PY)	Incidence rate (per 10^3 PY)	Age-adjusted HR (95% CI)	Multivariable-adjusted HR (95% CI)	
						Model 1[a]	Model 2[b]
Men	**(1) Age 18-29 years (n= 38,654)**						
	Q1	476	48,382.7	9.84	0.94 (0.83, 1.06)	0.81 (0.72, 0.92)	0.82 (0.73, 0.93)
	Q2	540	52,041.0	10.38	0.98 (0.81, 1.11)	0.94 (0.84, 1.06)	0.94 (0.84, 1.06)
	Q3	574	54,468.8	10.54	reference	reference	reference
	Q4	629	59,257.9	10.61	1.01 (0.90, 1.13)	1.03 (0.92, 1.15)	1.03 (0.92, 1.15)
	Q5	773	69,252.7	11.16	1.08 (0.97, 1.20)	1.13 (1.02, 1.26)	1.16 (1.04, 1.29)
	p for trend				0.016	<0.001	<0.001
	**(2) Age 30-39 years (n= 123,061)**						
	Q1	2,368	216,722.2	10.93	1.03 (0.97, 1.09)	0.89 (0.84, 0.94)	0.89 (0.84, 0.94)
	Q2	2,375	222,807.4	10.66	1.00 (0.95, 1.06)	0.97 (0.92, 1.03)	0.97 (0.92, 1.03)
	Q3	2,377	224,695.7	10.58	reference	reference	reference
	Q4	2,400	221,086.3	10.86	1.03 (0.98, 1.09)	1.07 (1.01, 1.14)	1.08 (1.02, 1.14)
	Q5	2,348	204,853.8	11.46	1.11 (1.05, 1.18)	1.21 (1.14, 1.28)	1.22 (1.15, 1.29)
	p for trend				0.006	<0.001	<0.001
	**(3) Age 40-49 years (n= 46,597)**						
	Q1	946	66,994.2	14.12	1.10 (1.00, 1.21)	0.92 (0.83, 1.01)	0.91 (0.83, 1.01)
	Q2	809	64,121.8	12.62	0.99 (0.90, 1.09)	0.95 (0.86, 1.05)	0.95 (0.86, 1.05)
	Q3	760	59,662.0	12.74	reference	reference	reference
	Q4	738	56,392.0	13.09	1.03 (0.93, 1.14)	1.09 (0.98, 1.20)	1.10 (0.99, 1.21)
	Q5	744	50,385.3	14.77	1.15 (1.01, 1.32)	1.28 (1.15, 1.41)	1.29 (1.17, 1.43)
	p for trend				0.398	<0.001	<0.001
Women	**(4) Age 18-29 years (n= 31,926)**						
	Q1	560	41,362.9	13.54	0.93 (0.83, 1.05)	0.89 (0.79, 1.00)	0.92 (0.82, 1.04)
	Q2	575	42,229.9	13.65	0.95 (0.85, 1.07)	0.93 (0.83, 1.04)	0.94 (0.84, 1.05)
	Q3	604	42,226.7	14.30	reference	reference	reference
	Q4	612	44,709.2	13.69	0.95 (0.85, 1.06)	0.95 (0.85, 1.07)	0.96 (0.86, 1.08)
	Q5	683	52,657.5	12.97	0.88 (0.79, 0.98)	0.89 (0.80, 0.99)	0.90 (0.81, 1.01)
	p for trend				0.296	0.944	0.758
	**(5) Age 30-39 years (n= 86,279)**						
	Q1	1,720	145,491.4	11.82	0.97 (0.91, 1.04)	0.94 (0.88, 1.01)	0.96 (0.90, 1.03)

Q2	1,711	139,415.0	12.27	1.01 (0.95, 1.08)	1.01 (0.94, 1.08)	1.02 (0.95, 1.09)
Q3	1,651	136,537.1	12.09	reference	reference	reference
Q4	1,688	134,988.2	12.50	1.04 (0.97, 1.11)	1.04 (0.97, 1.12)	1.04 (0.97, 1.11)
Q5	1,684	129,932.4	12.96	1.08 (1.01, 1.16)	1.07 (0.99, 1.14)	1.05 (0.98, 1.13)
p for trend				0.001	<0.001	0.022
**(6) Age 40-49 years (26,623)**						
Q1	381	31,488.5	12.10	1.01 (0.87, 1.16)	0.92 (0.80, 1.07)	0.93 (0.80, 1.08)
Q2	379	30,495.0	12.43	1.02 (0.89, 1.18)	1.01 (0.87, 1.16)	1.01 (0.87, 1.16)
Q3	384	31,483.0	12.20	reference	reference	reference
Q4	417	31,870.6	13.08	1.07 (0.93, 1.23)	1.10 (0.96, 1.26)	1.10 (0.96, 1.27)
Q5	341	29,657.7	11.50	0.94 (0.82, 1.09)	0.98 (0.85, 1.14)	0.98 (0.85, 1.14)
p for trend				0.672	0.210	0.248

Median (Q1-Q3) follow-up duration: (1) 6.41 (3.71–10.11) years, (2) 7.92 (3.79–12.65) years, (3) 5.03 (2.21–9.30) years, (4) 6.36 (3.72–9.75) years, (5) 7.01 (3.60–11.02) years, (6) 4.70 (2.11–8.13) years.

CKD: chronic kidney disease. TyG index: triglyceride and glucose index, eGFR: estimated Glomerular Filtration Rate, HOMA-IR: homeostasis model assessment of insulin resistance.

[a] Model 1 adjusted for age, examination center, body mass index, education, smoking status, alcohol intake, regular physical activity, history of diabetes, history of hypertension, usage of lipid-lowering medication, eGFR, TyG index, menopause (for women).

[b] Model 2 adjusted for variables in Model 1 and total cholesterol, high density lipoprotein cholesterol, low density lipoprotein cholesterol, HOMA-IR, uric acid.

Supplementary Table S5. Risk of incident CKD according to the quintiles of TyG index change by eGFR level

	Quintile	Events (n)	Duration (PY)	Incidence rate (per 10^3 PY)	Age-adjusted HR (95% CI)	Multivariable-adjusted HR (95% CI)	
						Model 1[a]	Model 2[b]
Men	**(1) eGFR ≥ 90 mL/min/1.73m² (n= 108,779)**						
	Q1	1,692	143,836.3	11.76	1.04 (0.97, 1.11)	0.87 (0.81, 0.93)	0.86 (0.81, 0.93)
	Q2	1,616	144,628.4	11.17	0.98 (0.92, 1.05)	0.95 (0.89, 1.02)	0.94 (0.88, 1.01)
	Q3	1,675	148,063.6	11.31	reference	reference	reference
	Q4	1,772	153,152.9	11.57	1.03 (0.96, 1.10)	1.09 (1.02, 1.16)	1.09 (1.02, 1.17)
	Q5	1,883	158,682.8	11.87	1.08 (1.01, 1.15)	1.19 (1.11, 1.27)	1.21 (1.13, 1.29)
	p for trend				0.091	<0.001	<0.001
	**(2) eGFR < 90 mL/min/1.73m² (n= 99,533)**						
	Q1	2,098	188,262.8	11.14	1.03 (0.97, 1.09)	0.90 (0.84, 0.95)	0.89 (0.84, 0.95)
	Q2	2,108	194,341.7	10.85	1.01 (0.95, 1.07)	0.97 (0.91, 1.03)	0.97 (0.91, 1.03)
	Q3	2,036	190,761.8	10.67	reference	reference	reference
	Q4	1,995	183,583.2	10.87	1.03 (0.96, 1.09)	1.06 (1.00, 1.13)	1.07 (1.00, 1.14)
	Q5	1,982	165,809.0	11.95	1.15 (1.08, 1.22)	1.23 (1.16, 1.31)	1.25 (1.17, 1.33)
	p for trend				0.002	<0.001	<0.001
Women	**(3) eGFR ≥ 90 mL/min/1.73m² (n= 93,554)**						
	Q1	1,560	111,093.9	14.04	0.97 (0.90, 1.04)	0.90 (0.84, 0.97)	0.94 (0.87, 1.01)
	Q2	1,578	111,375.6	14.17	0.98 (0.91, 1.05)	0.96 (0.90, 1.03)	0.97 (0.90, 1.04)
	Q3	1,640	113,495.8	14.45	reference	reference	reference
	Q4	1,701	116,913.1	14.55	1.01 (0.94, 1.08)	1.02 (0.95, 1.09)	1.03 (0.96, 1.10)
	Q5	1,763	125,835.2	14.01	0.96 (0.90, 1.03)	0.98 (0.91, 1.05)	0.99 (0.92, 1.06)
	p for trend				0.836	0.019	0.062
	**(4) eGFR < 90 mL/min/1.73m² (n= 51,274)**						
	Q1	1,101	107,248.8	10.27	0.99 (0.91, 1.08)	0.96 (0.88, 1.05)	0.98 (0.89, 1.07)
	Q2	1,087	100,654.3	10.80	1.04 (0.96, 1.14)	1.05 (0.96, 1.14)	1.05 (0.96, 1.15)
	Q3	999	96,751.0	10.33	reference	reference	reference
	Q4	1,016	94,654.9	10.73	1.04 (0.96, 1.14)	1.05 (0.96, 1.14)	1.04 (0.96, 1.14)
	Q5	945	86,412.3	10.94	1.07 (0.98, 1.17)	1.06 (0.97, 1.16)	1.04 (0.95, 1.14)
	p for trend				0.126	0.075	0.322

Median (Q1-Q3) follow-up duration: (1) 6.12 (3.07–9.88) years, (2) 8.04 (3.62–13.87) years, (3) 5.91 (3.00–9.01) years, (4) 8.31 (3.79–14.90) years.

CKD: chronic kidney disease. TyG index: triglyceride and glucose index, eGFR: estimated Glomerular Filtration Rate, HOMA-IR: homeostasis model

assessment of insulin resistance.
[a] Model 1 adjusted for age, examination center, body mass index, education, smoking status, alcohol intake, regular physical activity, history of diabetes, history of hypertension, usage of lipid-lowering medication, eGFR, TyG index, menopause (for women).
[b] Model 2 adjusted for variables in Model 1 and total cholesterol, high density lipoprotein cholesterol, low density lipoprotein cholesterol, HOMA-IR, uric acid.

Supplementary Table S6. Risk of incident CKD according to quintiles of TyG index change, stratified by regular physical activity status

	Quintile	Events (n)	Duration (PY)	Incidence rate (per 10^3 PY)	Age-adjusted HR (95% CI)	Multivariable-adjusted HR (95% CI)	
						Model 1[a]	Model 2[b]
Men	**(1) Vigorous exercise frequency<3 times/week (n= 177,966)**						
	Q1	3,344	291,057.4	11.49	1.03 (0.99, 1.08)	0.89 (0.85, 0.93)	0.88 (0.84, 0.93)
	Q2	3,254	297,601.6	10.93	0.99 (0.94, 1.03)	0.95 (0.91, 1.00)	0.95 (0.90, 1.00)
	Q3	3,268	296,807.3	11.01	reference	reference	reference
	Q4	3,252	291,111.1	11.17	1.02 (0.98, 1.07)	1.06 (1.01, 1.12)	1.07 (1.02, 1.12)
	Q5	3,244	171,880.8	11.93	1.12 (1.06, 1.17)	1.21 (1.15, 1.27)	1.23 (1.17, 1.29)
	p for trend				<0.001	<0.001	<0.001
	**(2) Vigorous exercise frequency≥3 times/week (n= 30,346)**						
	Q1	446	41,041.7	10.87	1.02 (0.90, 1.16)	0.86 (0.76, 0.99)	0.86 (0.75, 0.99)
	Q2	470	41,368.5	11.36	1.08 (0.95, 1.23)	1.04 (0.91, 1.18)	1.03 (0.91, 1.18)
	Q3	443	42,018.2	10.54	reference	reference	reference
	Q4	515	45,625.1	11.29	1.08 (0.95, 1.22)	1.12 (0.99, 1.28)	1.12 (0.98, 1.27)
	Q5	621	52,611.0	11.80	1.16 (1.02, 1.31)	1.27 (1.12, 1.43)	1.27 (1.12, 1.43)
	p for trend				0.061	<0.001	<0.001
Women	**(3) Vigorous exercise frequency<3 times/week (n= 129,659)**						
	Q1	2,384	196,377.2	12.14	0.95 (0.90, 1.00)	0.91 (0.86, 0.97)	0.94 (0.88, 0.99)
	Q2	2,403	190,465.3	12.62	0.99 (0.94, 1.05)	0.98 (0.93, 1.04)	0.99 (0.94, 1.05)
	Q3	2,390	188,403.5	12.69	reference	reference	reference
	Q4	2,431	189,084.3	12.86	1.01 (0.96, 1.07)	1.02 (0.97, 1.08)	1.03 (0.97, 1.08)
	Q5	2,380	186,629.6	12.75	1.01 (0.95, 1.06)	1.00 (0.94, 1.06)	1.00 (0.94, 1.06)
	p for trend				0.032	0.003	0.029
	**(4) Vigorous exercise frequency≥3 times/week (n= 15,169)**						
	Q1	277	21,965.5	12.61	1.11 (0.94, 1.32)	1.07 (0.90, 1.27)	1.09 (0.92, 1.30)
	Q2	262	21,564.6	12.15	1.08 (0.90, 1.29)	1.07 (0.90, 1.27)	1.07 (0.90, 1.28)
	Q3	249	21,843.3	11.40	reference	reference	reference
	Q4	286	22,483.7	12.72	1.12 (0.95, 1.33)	1.12 (0.94, 1.33)	1.11 (0.94, 1.31)
	Q5	328	25,618.0	12.80	1.13 (0.95, 1.33)	1.12 (0.95, 1.33)	1.09 (0.92, 1.29)
	p for trend				0.695	0.444	0.844

Median (Q1-Q3) follow-up duration: (1) 7.02 (3.42–11.64) years, (2) 6.05 (2.87–10.62) years, (3) 6.27 (3.21–10.16) years, (4) 6.25 (3.10–10.44) years.

CKD: chronic kidney disease. TyG index: triglyceride and glucose index, eGFR: estimated Glomerular Filtration Rate, HOMA-IR: homeostasis model

assessment of insulin resistance.

[a] Model 1 adjusted for age, examination center, body mass index, education, smoking status, alcohol intake, history of diabetes, history of hypertension, usage of lipid-lowering medication, eGFR, TyG index, menopause (for women).

[b] Model 2 adjusted for variables in Model 1 and total cholesterol, high density lipoprotein cholesterol, low density lipoprotein cholesterol, HOMA-IR, uric acid.

Supplementary Table S7. Risk of incident CKD according to quintiles of TyG index change, stratified by alcohol intake status

	Quintile	Events (n)	Duration (PY)	Incidence rate (per 10^3 PY)	Age-adjusted HR (95% CI)	Multivariable-adjusted HR (95% CI)	
						Model 1[a]	Model 2[b]
Men	**(1) Alcohol intake<20g/day (n= 158,128)**						
	Q1	2,752	250,887.6	10.97	1.03 (0.98, 1.09)	0.89 (0.85, 0.94)	0.89 (0.85, 0.94)
	Q2	2,759	265,176.6	10.40	0.98 (0.93, 1.03)	0.95 (0.90, 1.00)	0.95 (0.90, 1.00)
	Q3	2,805	265,988.6	10.55	reference	reference	reference
	Q4	2,788	263,507.0	10.58	1.01 (0.96, 1.07)	1.05 (0.99, 1.10)	1.05 (1.00, 1.11)
	Q5	2,739	246,968.0	11.09	1.08 (1.03, 1.14)	1.18 (1.12, 1.24)	1.19 (1.13, 1.26)
	p for trend				0.031	<0.001	<0.001
	**(2) Alcohol intake≥20g/day (n= 50,184)**						
	Q1	1,038	81,211.5	12.78	1.02 (0.94, 1.12)	0.87 (0.79, 0.95)	0.86 (0.76, 0.94)
	Q2	965	73,793.5	13.08	1.05 (0.96, 1.15)	1.01 (0.92, 1.10)	1.00 (0.92, 1.10)
	Q3	906	72,836.8	12.44	reference	reference	reference
	Q4	979	73,229.2	13.37	1.08 (0.99, 1.19)	1.14 (1.04, 1.25)	1.15 (1.05, 1.26)
	Q5	1,126	77,523.8	14.52	1.20 (1.10, 1.31)	1.33 (1.22, 1.45)	1.35 (1.24, 1.48)
	p for trend				<0.001	<0.001	<0.001
Women	**(3) Alcohol intake<10g/day (n= 128,129)**						
	Q1	2,369	198,375.7	11.94	0.98 (0.92, 1.04)	0.94 (0.89, 1.00)	0.97 (0.91, 1.03)
	Q2	2,369	192,751.1	12.29	1.01 (0.96, 1.07)	1.01 (0.95, 1.07)	1.02 (0.96, 1.08)
	Q3	2,302	190,034.4	12.11	reference	reference	reference
	Q4	2,370	191,017.0	12.41	1.02 (0.97, 1.09)	1.03 (0.97, 1.09)	1.03 (0.98, 1.09)
	Q5	2,337	188,766.8	12.38	1.02 (0.98, 1.08)	1.01 (0.96, 1.08)	1.01 (0.95, 1.07)
	p for trend				0.106	0.024	0.163
	**(4) Alcohol intake≥10g/day (n= 16,609)**						
	Q1	292	19,967.1	14.62	0.88 (0.75, 1.02)	0.80 (0.68, 0.94)	0.82 (0.69, 0.96)
	Q2	296	19,278.8	15.35	0.93 (0.79, 1.08)	0.89 (0.77, 1.05)	0.90 (0.77, 1.05)
	Q3	337	20,212.4	16.67	reference	reference	reference
	Q4	347	20,551.0	16.88	1.01 (0.87, 1.17)	1.02 (0.88, 1.19)	1.02 (0.88, 1.19)
	Q5	371	23,480.7	15.80	0.95 (0.82, 1.10)	0.98 (0.84, 1.13)	0.97 (0.84, 1.13)
	p for trend				0.188	0.007	0.016

Median (Q1-Q3) follow-up duration: (1) 6.97 (3.37–11.70) years, (2) 6.58 (3.14–10.91) years, (3) 6.41 (3.26–10.37) years, (4) 5.83 (2.92–8.89) years.

CKD: chronic kidney disease. TyG index: triglyceride and glucose index, eGFR: estimated Glomerular Filtration Rate, HOMA-IR: homeostasis model

assessment of insulin resistance.

[a] Model 1 adjusted for age, examination center, body mass index, education, smoking status, regular physical activity, history of diabetes, history of hypertension, usage of lipid-lowering medication, eGFR, TyG index, menopause (for women).

[b] Model 2 adjusted for variables in Model 1 and total cholesterol, high density lipoprotein cholesterol, low density lipoprotein cholesterol, HOMA-IR, uric acid.

Supplementary Table S8. Risk of incident CKD according to quintiles of TyG index change, stratified by HOMA-IR group

	Quintile	Events (n)	Duration (PY)	Incidence rate (per 10^3 PY)	Age-adjusted HR (95% CI)	Multivariable-adjusted HR (95% CI)	
						Model 1[a]	Model 2[b]
**Men**	**(1) Low HOMA-IR group (<75th percentile) (n= 155,963)**						
	Q1	2,196	225,191.0	9.75	1.02 (0.97, 1.09)	0.94 (0.89, 1.00)	0.94 (0.89, 1.00)
	Q2	2,384	249,121.3	9.57	1.01 (0.95, 1.07)	0.98 (0.93, 1.04	0.98 (0.93, 1.03)
	Q3	2,431	257,650.5	9.44	reference	reference	reference
	Q4	2,597	265,377.2	9.79	1.05 (0.99, 1.11)	1.07 (1.01, 1.13	1.07 (1.01, 1.13)
	Q5	2,820	268,604.5	10.50	1.15 (1.09, 1.22)	1.20 (1.14, 1.27)	1.22 (1.15, 1.29)
	p for trend				<0.001	<0.001	<0.001
	**(2) High HOMA-IR group (≥75th percentile) (n= 52,349)**						
	Q1	1,594	106,908.1	14.91	0.94 (0.87, 1.01)	0.80 (0.75, 0.87)	0.80 (0.75, 0.87)
	Q2	1,340	89,848.8	14.91	0.94 (0.87, 1.02)	0.93 (0.86, 1.000)	0.93 (0.86, 1.00)
	Q3	1,280	81,264.9	15.75	reference	reference	reference
	Q4	1,170	71,359.0	16.40	1.05 (0.97, 1.134)	1.08 (1.00, 1.17)	1.09 (1.01, 1.18)
	Q5	1,045	55,887.3	18.70	1.21 (1.11, 1.310)	1.26 (1.16, 1.37)	1.27 (1.17, 1.37)
	p for trend				<0.001	<0.001	<0.001
**Women**	**(3) Low HOMA-IR group (<75th percentile) (n= 108,209)**						
	Q1	1,658	140,283.7	11.82	0.94 (0.88, 1.005)	0.94 (0.88, 1.01)	0.97 (0.90, 1.03)
	Q2	1,861	151,215.4	12.31	0.99 (0.93, 1.052)	0.99 (0.93, 1.05)	1.00 (0.94, 1.07)
	Q3	1,955	157,296.5	12.43	reference	reference	reference
	Q4	2,077	164,280.4	12.64	1.02 (0.96, 1.081)	1.02 (0.96, 1.08)	1.02 (0.96, 1.08)
	Q5	2,188	168,535.3	12.57	1.01 (0.95, 1.078)	1.01 (0.95, 1.07)	1.00 (0.94, 1.06)
	p for trend				0.019	0.053	0.381
	**(4) High HOMA-IR group (≥75th percentile) (n= 36,619)**						
	Q1	1,003	78,069.1	12.85	0.99 (0.90, 1.094)	0.91 (0.82, 1.01)	0.93 (0.84, 1.03)
	Q2	804	60,814.5	13.22	1.02 (0.92, 1.131)	1.01 (0.92, 1.12)	1.02 (0.92, 1.13)
	Q3	684	52,950.3	12.92	reference	reference	reference
	Q4	640	47,287.6	13.53	1.05 (0.94, 1.166)	1.06 (0.95, 1.18)	1.06 (0.95, 1.18)
	Q5	590	43,712.2	13.50	1.04 (0.93, 1.160)	1.01 (0.90, 1.13)	1.01 (0.91, 1.13)
	p for trend				0.315	0.024	0.054

Median (Q1-Q3) follow-up duration: (1) 7.14 years (3.55–11.59) years, (2) 6.04 (2.90–11.03) years, (3) 6.34 (3.24–10.10) years, (4) 6.10 (3.09–10.66) years.

CKD: chronic kidney disease. TyG index: triglyceride and glucose index, eGFR: estimated Glomerular Filtration Rate, HOMA-IR: homeostasis model

assessment of insulin resistance.

[a] Model 1 adjusted for age, examination center, body mass index, education, smoking status, regular physical activity, history of diabetes, history of hypertension, usage of lipid-lowering medication, eGFR, TyG index, menopause (for women).

[b] Model 2 adjusted for variables in Model 1 and total cholesterol, high density lipoprotein cholesterol, low density lipoprotein cholesterol, HOMA-IR, uric acid.

Supplementary Table S9. Discrimination ability of TyG index change for chronic kidney disease prediction by time horizon

	At 5-years	At 10-years	At 15-years
**Men**			
AUC	0.494	0.515	0.527
Std. Error	0.003	0.003	0.003
95% CI	0.488, 0.500	0.510, 0.520	0.521, 0.534
p-value	0.071	<0.001	<0.001
Optimal cutoffs	0.716	0.372	0.263
J-Youden	0.012	0.031	0.047
Sensitivity (%)	7.9%	23.9%	31.6%
Specificity (%)	93.3%	79.3%	73.0%
(+) Likelihood ratio	1.177	1.152	1.172
(−) Likelihood ratio	0.987	0.960	0.936
**Women**			
AUC	0.493	0.517	0.541
Std. Error	0.004	0.003	0.004
95% CI	0.485, 0.500	0.511, 0.523	0.533, 0.549
p-value	<0.001	<0.001	<0.001
Optimal cutoffs	-0.144	-0.145	0.068
J-Youden	0.005	0.026	0.056
Sensitivity (%)	68.8%	69.3%	47.9%
Specificity (%)	55.4%	57.3%	57.7%
(+) Likelihood ratio	1.542	1.625	1.132
(−) Likelihood ratio	0.564	0.535	0.903

Standard errors and 95% confidence intervals of AUCs were estimated using 200 bootstrap resamples.

Abbreviations. AUC, area under the curve; Std. Error, standard error; CI, confidence interval.

Supplementary Table S10. Risk of incident chronic kidney disease according to quintiles of triglyceride-glucose (TyG) index percent change

Quintile	N	Events (N)	Duration (PY)	Incidence rate (per 10^3 PY)	Age-adjusted HR (95% CI)	Multivariable-adjusted HR (95% CI)	
						Model 1[a]	Model 2[b]
Men							
Q1 (-32.6% – -3.37%)	41,663	3,736	332,107.6	11.25	1.00 (0.95, 1.04)	0.88 (0.84, 0.92)	0.88 (0.84, 0.92)
Q2 (-3.37% – -0.55%)	41,662	3,755	338,771.2	11.08	0.98 (0.94, 1.03)	0.95 (0.91, 1.00)	0.95 (0.91, 0.99)
Q3 (-0.55% – 1.87%)	41,662	3,805	338,713.3	11.23	reference	reference	reference
Q4 (1.87% – 4.86%)	41,662	3,784	337,174.5	11.22	1.01 (0.96, 1.01)	1.06 (1.01, 1.11)	1.06 (1.02, 1.11)
Q5 (4.86% – 42.10%)	41,663	3,777	324,356.0	11.64	1.07 (1.03, 1.12)	1.21 (1.16, 1.27)	1.23 (1.17, 1.29)
p for trend					<0.001	<0.001	<0.001
Women							
Q1 (-34.0% – -3.59%)	28,966	2,654	218,140.5	12.17	0.96 (0.91, 1.01)	0.92 (0.87, 0.98)	0.95 (0.89, 1.00)
Q2 (-3.59% – -0.74%)	28,965	2,667	212068.8	12.58	0.99 (0.94, 1.05)	0.99 (0.94, 1.04)	0.99 (0.94, 1.05)
Q3 (-0.74% – 1.75%)	28,966	2,657	210,490.2	12.62	reference	reference	reference
Q4 (1.75% – 4.8%)	28,965	2,712	211,810.3	12.80	1.01 (0.96, 1.07)	1.02 (0.97, 1.08)	1.02 (0.97, 1.08)
Q5 (4.8% – 47.5%)	28,966	2,700	211,925.2	12.74	1.01 (0.96, 1.07)	1.01 (0.96, 1.07)	1.01 (0.95, 1.06)
p for trend					0.035	0.002	0.030

Median (Q1-Q3) follow-up duration: 6.90 (3.30–11.46) years for men and 6.27 (3.20–10.18) years for women.

Abbreviations. CI, confidence interval; eGFR, estimated Glomerular Filtration Rate, HR, hazard ratio; HOMA-IR, homeostasis model assessment of insulin resistance; PY, person-year; Q, quintile.

[a] Model 1 adjusted for age, examination center, body mass index, education, smoking status, alcohol intake, regular physical activity, history of diabetes, history of hypertension, usage of lipid-lowering medication, eGFR, TyG index, menopause (for women).
[b] Model 2 adjusted for variables in Model 1 and total cholesterol, high-density lipoprotein cholesterol, low-density lipoprotein cholesterol, HOMA-IR, and uric acid.

Supplementary Table S11. Interaction between TyG index change quintiles and sex in incident chronic kidney disease

	HR (95% CI)	*p*-value
Interaction terms (TyG index quintiles × Sex)		
Q1 × Men	1.10 (1.03, 1.18)	0.006
Q2 × Men	1.01 (0.94, 1.09)	0.749
Q4 × Men	1.00 (0.93, 1.07)	0.947
Q5 × Men	1.10 (1.03, 1.18)	0.007
p for interaction	<0.001	

Reference: Women in Q3.

p for interaction was obtained from a likelihood ratio test comparing full adjusted multivariable Cox models with and without the interaction term.

Abbreviations. HR, hazard ratio; CI, confidence interval; Q, quintile.

Supplementary Figure Legends

Supplementary Figure S1. Restricted cubic spline curves for the association between changes in the triglyceride–glucose (TyG) index and the risk of incident chronic kidney disease (CKD). Hazard ratios (solid lines) and 95% confidence intervals (shaded areas) were estimated using Cox proportional hazards models with knots at the 5th, 27.5th, 50th, 72.5th, and 95th percentiles of the TyG index change distribution. The models were adjusted for covariates, as described in the Materials and Methods section.

Supplementary Figure S2. Receiver operating characteristic (ROC) curves of men (left) and women (right) at 5, 10, and 15-years. Abbreviations. AUC, area under the curve.

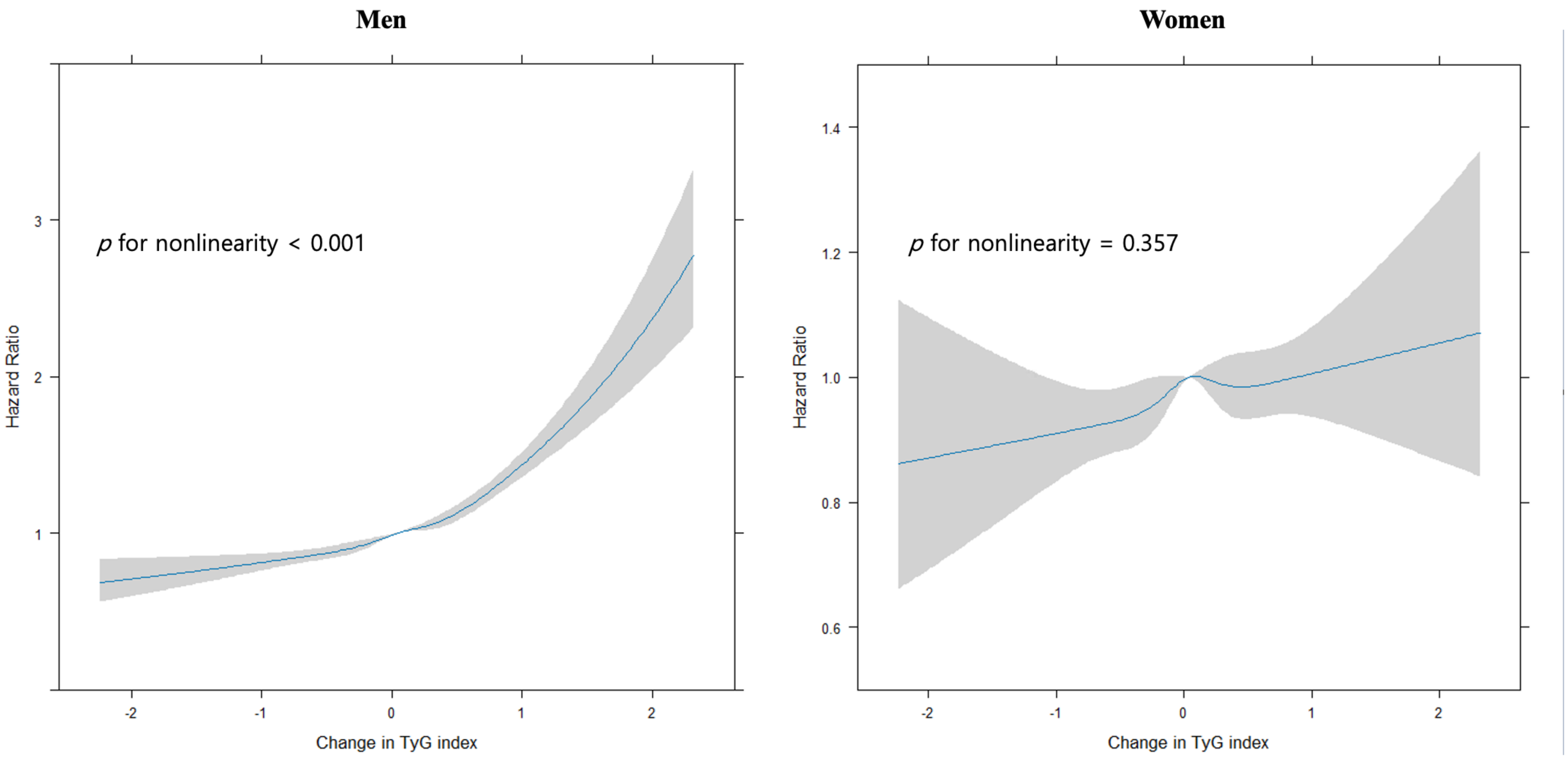

Supplementary Figure S1.

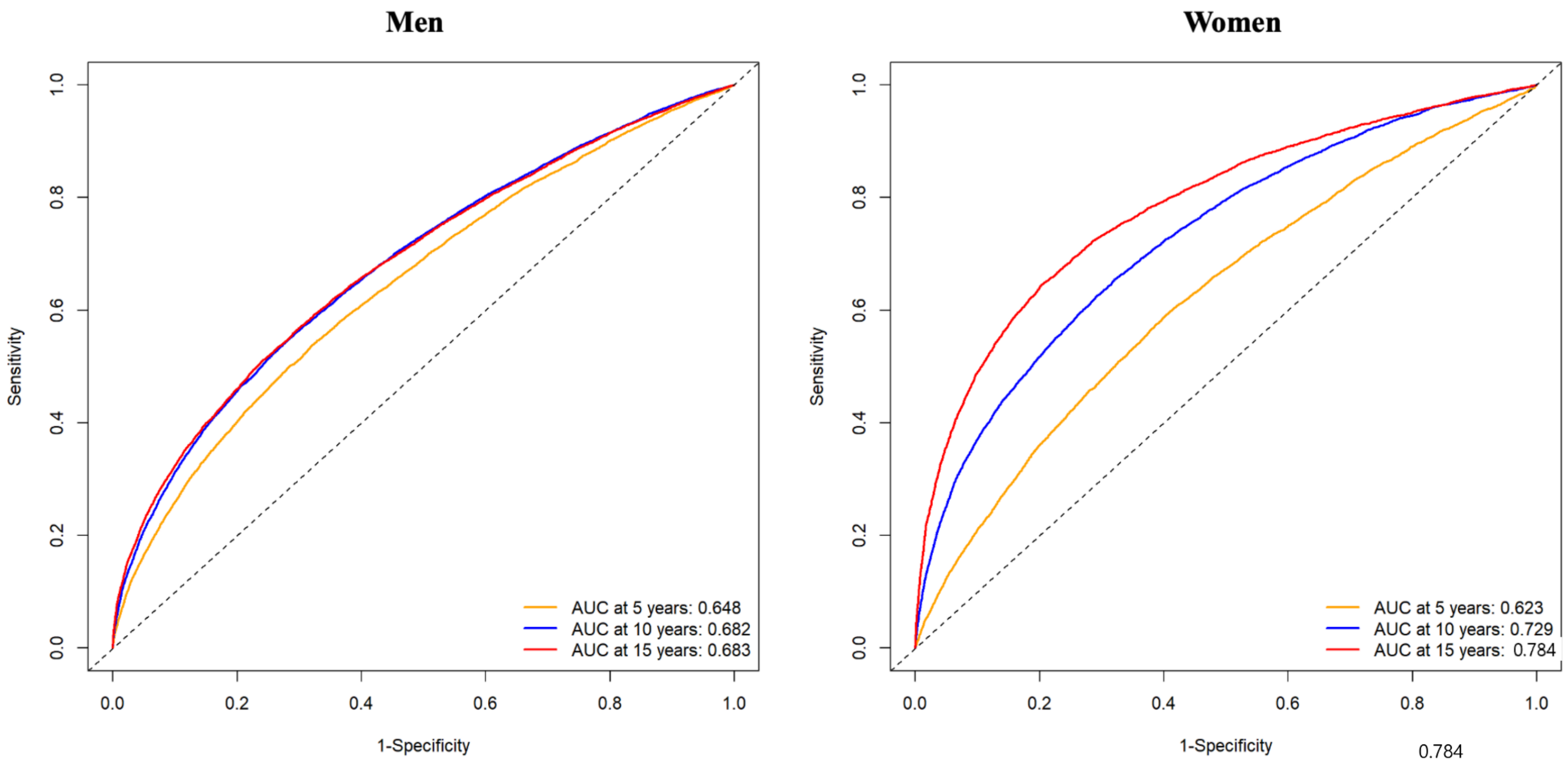

Supplementary Figure S2.
